# An Effective Cationic Human Serum Albumin-Based Gene-Delivery Carrier Containing the Nuclear Localization Signal

**DOI:** 10.3390/pharmaceutics11110608

**Published:** 2019-11-13

**Authors:** Guannan Guan, Baohui Song, Jie Zhang, Kang Chen, Haiyang Hu, Mingyue Wang, Dawei Chen

**Affiliations:** 1 Department of Pharmaceutics, School of Pharmacy, Shenyang Pharmaceutical University, No. 103, Wenhua Road, Shenyang 110016, China; pharmggn@gmail.com (G.G.); sbh940101@aliyun.com (B.S.); huhaiyang@syphu.edu.cn (H.H.); 2 Department of Pharmaceutics, Medical College of Jiaxing University, Jiaxing 314001, China; zhangjiejidi@outlook.com; 3 Department of Medicine, The University of Hong Kong, 21 Sassoon Road, Hong Kong 999077, China; kcjackie@hku.hk; 4 Department of Pharmacy, Shenyang Medical College, No. 146, Huanghe North Street, Shenyang 110034, China

**Keywords:** cationic human serum albumin, p53 plasmid, nuclear locating sequences, antitumor activity, gene delivery

## Abstract

Considerable effort has been devoted to the development of gene carriers over the years. However, toxicity, immunogenicity, and low transfection efficiency are still major barriers. How to overcome these obstacles has become a burning question in gene delivery. In the present study, a simple cationic human serum albumin (CHSA)-based gene-delivery system containing nuclear localization signals (NLSs) was constructed to conquer the limitations. CHSA/NLS/plasmid DNA (pDNA) complexes were prepared and characterized by Hoechst 33258 intercalation, gel retardation assay, morphological analysis, circular dichroism (CD) spectroscopy, particle size, and zeta potential measurements. Results showed that CHSA/NLS/pDNA complexes were able to condense and protect pDNA with high encapsulation efficiency. The complexes displayed a nutritional effect on cells at a low concentration and there was no significant cytotoxicity or immunogenicity. In addition, CHSA/NLS/pDNA complexes exhibited excellent cellular uptake rates and the mechanism was mainly the clathrin or macropinocytosis-dependent endocytosis pathway. Furthermore, CHSA/NLS/pDNA significantly enhanced gene expression efficiency in vitro. More importantly, CHSA/NLS/pDNA complexes showed a desired antitumor effect in vivo, exhibiting the highest inhibition rate (57.3%) and significant upregulation in p53 protein. All these results confirm that CHSA/NLS/pDNA complexes have a bright future as a safe and effective delivery system for gene therapy.

## 1. Introduction

Over the past several decades, gene therapy has become one of the most actively developing and promising branches of medicine. The application of gene-based therapy to treat or prevent a wide range of diseases has been investigated [1,2,3]. However, success in clinical trials has been restricted due to numerous technical barriers such as low safety and high immunogenicity as well as limited transfection efficiency [4].

Human serum albumin (HSA), an attractive macromolecular carrier for drug delivery, has been shown to be nontoxic, nonimmunogenic, biocompatible, stable in plasma, metabolized in vivo to produce innocuous degradation products, easy to purify, and soluble in water, allowing ease of delivery by injection, and thus represents an ideal candidate for fabrication of nanoparticles for drug and gene delivery [5,6]. Human serum albumin is extremely robust to pH, temperature, and organic solvents. Due to the defined primary structure and high content of charged amino acids (e.g., lysine), human serum albumin-based nanoparticles allow for the electrostatic adsorption of negatively charged molecules such as plasmid DNA (pDNA) without requiring other compounds [7]. Furthermore, HSA-based nanocarriers appear to be suitable agents for gene delivery because they might avoid the undesired interactions with serum that are often encountered after intravenous injection of transfection complexes; more importantly, they are known to accumulate in tumors, being preferentially taken up by tumor cells at increased levels compared to normal cells [8,9].

However, the application of HSA-based nanocarriers in gene delivery is limited because of restricted encapsulation and protection of genetic material as well as variable transfection rates when injected intravenously [10,11]. An effective solution to this problem is to modify HSA by cationization. Compared to HSA, cationic human serum albumin may be a more effective nanocarrier for gene delivery due to its ability to electrostatically condense negatively charged nucleic acids into weakly cationic nanoparticles that penetrate the anionic cell membrane more effectively [12]. Nanoparticles consisting of DNA, human serum albumin, and polyethyleneimine (HSA–PEI–DNA nanoparticles) were formed as a nonviral gene-delivery vehicle, and the data showed that serum albumin increased PEI-mediated gene transfer into airway epithelial CFT1-C2 cells and human embryonic epithelial kidney 293 cells in vitro [13,14]. However, applying this strategy to different pDNA is not convenient [15]. In addition, this strategy has not been used in therapeutic plasmid DNA and the uptake mechanism of the carrier is not yet clear. Moreover, there have been no results showing whether cationic HSA could increase the transfection and gene expression effect in vivo.

Gene expression of exogenous DNA depends on an essential nuclear importation process. Since free plasmid is restricted out of the nucleus, it needs to be improved in order to utilize nuclear localization sequences to facilitate the active transport of plasmid DNA into the nucleus from the cytoplasm through binding to nuclear transport proteins, thus endowing high gene expression [16]. Simian virus 40 (SV40) large tumor antigen (T-antigen) is a widely studied and commonly investigated nuclear localization signal (NLS) for the delivery of macromolecules with a well-defined nuclear import pathway [17]. 

**Figure 1:**
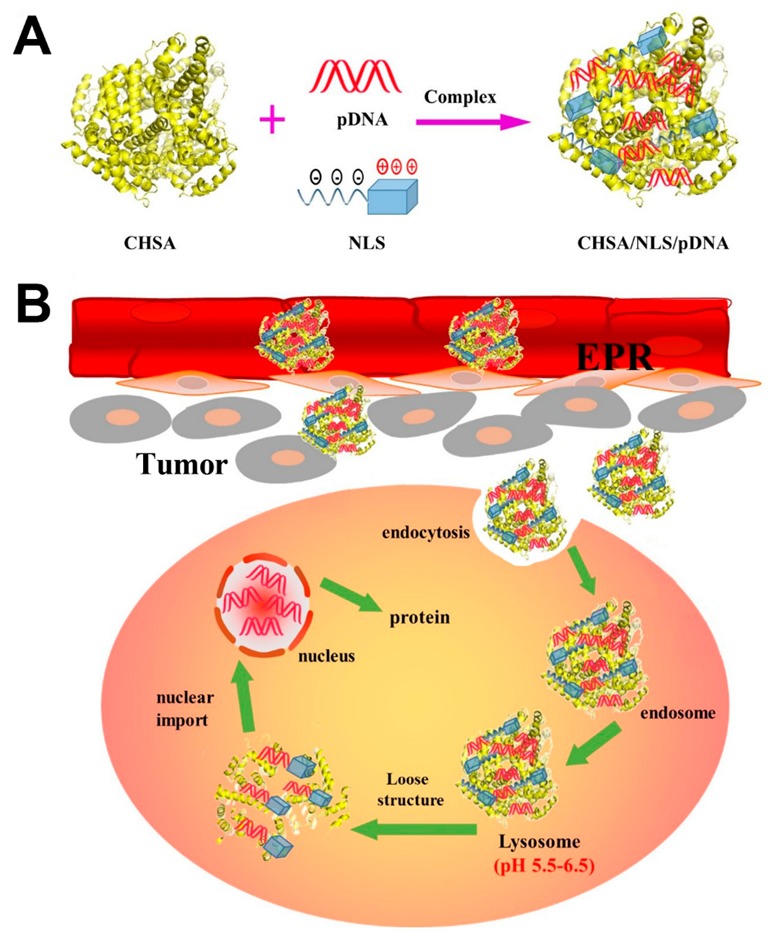
Schematic diagram showing (**A**) the formation of cationic human serum albumin (CHSA)/nuclear localization signal (NLS)/plasmid DNA (pDNA) complexes and (**B**) extracellular and intracellular trafficking for systemic delivery of plasmid DNA to tumor cells. Complexes accumulate in the tumor via the enhanced permeability and retention (EPR) effect and are associated with the tumor cell surface, followed by cellular endocytosis. Then the pDNA is delivered to the nucleus with the help of NLS due to its nuclear locating ability.

In this study, we constructed a simple and effective cationic human serum albumin (CHSA)-based gene-delivery system for CHSA/NLS/pDNA complexes. HSA-based carrier has shown remarkable promise for anticancer agents because HSA is a blood-compatible protein and is not immunogenic. HSA-based carrier can prolong the circulation half-life of otherwise rapidly cleared drugs and, importantly, promote their accumulation within tumors [18,19]. In order to deliver plasmid DNA into the nuclei of cancer cells and obtain high gene expression, SV40 large T-antigen–derived NLS was utilized during the preparation of complexes. CHSA/NLS/pDNA complexes have several advantageous functions, including plasmid DNA protection, tumor cell targeting, lysosomal escape, and nuclear localization. This ingenious design may provide effective gene delivery to cancer cells and a high tumor inhibition rate. The formation of CHSA/NLS/pDNA complexes as well as extracellular and intracellular trafficking for the systemic delivery of plasmid DNA to tumor cells is illustrated in Figure 1. 

CHSA was synthesized through the surface modification of HSA and characterized by sodium dodecyl sulfate polyacrylamide gel electrophoresis(SDS-PAGE), FTIR spectroscopy, and circular dichroism (CD). CHSA/NLS/pDNA complexes at various *w*/*w* ratios of CHSA to pDNA were prepared via electrostatic interaction and characterized by Hoechst 33258 intercalation, gel retardation assay, morphological analysis, CD spectroscopy, particle size, and zeta potential measurements. Furthermore, in vitro and in vivo safety as well as the gene-delivery ability of the complexes were investigated. More importantly, in vivo antitumor activity of CHSA/NLS/pDNA complexes containing the tumor suppressor p53 gene were investigated to determine the in vivo antitumor effect. All the results have demonstrated that CHSA/NLS/pDNA complexes are a safe and effective delivery system for plasmid DNA.

## 2. Materials and Methods 

### 2.1. Materials

Human serum albumin was obtained from Thermo Scientific (Waltham, MA, USA); 3-(4,5-dimethylthiazol-2-yl)-2,5-diphenyl tetrazolium bromide (MTT), trypsin, dimethyl sulfoxide(DMSO), fluorescein isothiocyanate(FITC), and 1-ethyl-(3-3-dimethylaminopropyl) carbodiimide hydrochloride (EDC) were purchased from Sigma-Aldrich (St Louis, MO, USA). pcDNA3.0-HA-p53 was obtained from Fenghui Biotechnology (Beijing, China). Plasmid pEGFP-C1 was a gift from Professor Xiaojun Shi of Tsinghua University. NLS peptide of the SV40 large T-antigen (CGGGPKKKRKVED) and a scrambled sequence (NLS (scr), CGGGPKTKRKVED) were synthesized and purified by GenScript Corp. (Shanghai, China). HepG2 cells and A549 cells were obtained from the cell bank of the Chinese Academy of Sciences (Shanghai, China). Dulbecco’s modified Eagle’s medium (DMEM), fetal bovine serum (FBS), and penicillin–streptomycin (P/S) were purchased from Gibco (Grand Island, NY, USA). Hoechst 33258 was purchased from Beyotime (Haimen, China). The luciferase reporter gene assay kit and plasmid pGL3-control were obtained from Promega (Madison, WI, USA). Lipofectamine 2000, protein molecular weight maker, and Hypersensitive ECL luminescent fluid were purchased from Thermo Fisher (Waltham, MA, USA); agarose and ethidium bromide (EB) were purchased from Biowest and Invitrogen Corp., respectively. β-actin primary antibodies and corresponding horseradish peroxidase (HRP)-conjugated secondary antibodies were purchased from Abcam (Cambridge, UK). All other buffer solution components and chemicals were commercially available reagents of analytical grade.

Male BALB/c nude mice (18–22 g) were obtained from the Department of Experimental Animals, Shenyang Pharmaceutical University (Shenyang, China). All mice were housed in the SPF II lab. All animal experiments were carried out in accordance with guidelines evaluated and approved by the ethics committee of Shenyang Pharmaceutical University (SYPU-IACUC-C2018-12-14-102/SYPU-IACUC-C2019-3-20-109, Animal ethics committee of shenyang pharmaceutical university; 14 December 2018/20 March 2019).

### 2.2. Preparation and Characterization of Cationic Human Serum Albumin

Human serum albumin was modified by ethylene diamine to increase its isoelectric point. In brief, HSA was dissolved in distilled water, 60 mL of 2 M ethylene diamine was added slowly to 10 mL of 20% (*w*/*v*) HSA solution, and the pH was adjusted to 4.75 with concentrated HCl, and then EDC was added to the solution. After stirring at room temperature for 4 h, the reaction was terminated by 70 mL of 4 M acetic acid buffer (pH 4.75). The obtained cationic human serum albumin was concentrated and purified at 4 °C using dialysis assay and cation exchange chromatography. The collected CHSA was concentrated at low temperature and freeze-dried for use. 

The obtained CHSA was characterized by SDS-PAGE electrophoresis (Tanon, Shanghai, China), FTIR spectroscopy (Bruker, Faellanden, Switzerland), and circular dichroism (CD). The cationization degree of CHSA was determined by the trinitrobenzene sulfonic acid (TNBS) method according to previous reports [20,21]. The zeta potential at different pH values of CHSA with varying degrees of cationization was determined using a dynamic light scattering instrument (Zetasizer NanoZS, Malvern, UK), and the isoelectric point of different CHSA was obtained according to the pH–zeta potential curve.

### 2.3. Preparation and Characterization of CHSA/NLS/pDNA Complexes 

#### 2.3.1. Preparation of CHSA/NLS/pDNA Complexes

NLS (SV40 large T-antigen) was mixed with pDNA at a molar ratio of 1:1 in phosphate buffer solution (PBS, pH 7.4) for 30 min at room temperature. Then NLS/pDNA complexes containing 1.5 µg pDNA were incubated with CHSA at different concentrations for 30 min. CHSA-2/pDNA, CHSA-3/pDNA, CHSA-2/NLS/pDNA, and CHSA-3/NLS/pDNA at a series of *w*/*w* ratios (weight ratio of CHSA to pDNA) were prepared.

#### 2.3.2. Hoechst 33258 Intercalation Assay

The DNA condensation efficiency of nanocomplexes formed at different *w*/*w* ratios were analyzed using a Hoechst 33258 intercalation assay. In brief, 100 μL of CHSA/NLS/pDNA complex (containing 500 ng of DNA) at different *w*/*w* ratios was mixed with 100 μL of Hoechst 33258 solution (0.2 μg/mL) and incubated for 5 min at 37 °C. The fluorescence intensity was measured at 352 nm (ex) and 457 nm (em). The fluorescence intensity of free pDNA was set as the control. The encapsulation efficiency was calculated according to Equation (1):

Encapsulation efficiency (EE%) = (Flucontrol − Flusample)/Flucontrol × 100%(1)

#### 2.3.3. Gel Retardation Assay

Resistance to heparin replacement and protection ability against DNase I degradation of CHSA/NLS/pDNA complexes were examined using agarose gel electrophoresis. CHSA/NLS/pDNA complex at different *w*/*w* ratios was mixed with heparin solution and incubated for 0.5 h at 37 °C, then the samples were electrophoresed. In addition, 2 μL of CHSA/NLS/pDNA complex at various *w*/*w* ratios was incubated with 10 μL of the DNase I digestion system (DNase I 10 U, 50 mM KCL, 10 mM Tris-HCL, 10 mM MgCl_2_, 0.1% TritonX-100, pH 9.0) at 37 °C for 10 min. The mixtures were then incubated with 5 μL termination solution (400 mM NaCl, 100 mM EDTA, pH 8.0) for 5 min at room temperature. Then, 10 μL supernatant mixed with 2 μL loading buffer was loaded into a 0.7% agarose gel. Free NLS/pDNA complexes were used as the control. The gel was stained with ethidium bromide and photographed using a Tanon 2500R image system (Tanon, Shanghai, China).

#### 2.3.4. Particle Size and Zeta Potential Measurements and Morphological Analysis

The particle size and zeta potential of nanocomplexes were determined by a Zetasizer Nano-ZS dynamic light scattering spectrometer (Malvern, UK). In addition, the morphology of CHSA/NLS/pDNA complexes was visually observed via transmission electron microscopy (TEM; FEI TecnaiG220, FEI, Hillsboro, OR, USA).

#### 2.3.5. Circular Dichroism Spectroscopy

CHSA/NLS/pDNA complexes were diluted with double distilled water and CD spectroscopy analysis was carried out on a MOS-450 spectropolarimeter (Bio-Logic, Seyssinet-Pariset, France). The spectra were collected from 190 nm to 250 nm in a quartz cell of 1 mm path length at 25 °C in a nitrogen atmosphere. Double distilled water was used as the control.

### 2.4. MTT Assay

In order to investigate the cytotoxicity of CHSA/NLS/pDNA complexes, reporter plasmid pEGFP-C1 was condensed by CHSA and NLS with a series of *w*/*w* ratios. The toxic effects on HepG2 and A549 cells were evaluated by MTT assay after treating CHSA-3/NLS/pDNA complexes with different concentrations of CHSA-3 (1 μg/mL to 2 mg/mL) for 24 h, 48 h, and 72 h. In order to explore the influence of the *w*/*w* ratio on cytotoxicity, HepG2 and A549 cells were treated with CHSA-2/NLS/pDNA and CHSA-3/NLS/pDNA complexes at various *w*/*w* ratios for 48 h.

HepG2 cells and A549 cells were cultured in DMEM supplemented with 10% FBS and 1% antibiotics (penicillin/streptomycin) at 37 °C in a humidified atmosphere containing 5% CO_2_. HepG2 cells and A549 cells were seeded in 96-well plates (1 × 10^4^ cells/well) and cultured overnight. Cells were treated with: (a) CHSA-3/NLS/pDNA and CHSA-2/NLS/pDNA complexes at different *w*/*w* ratios and (b) CHSA-3 at various concentrations. After cells were incubated, 10 μL MTT (5 mg/mL) was added to each well. The cells were further incubated for 4 h, then 150 μL DMSO was added to dissolve the formazan crystals that formed in the live cells. Absorbance of the samples was measured at 490 nm and cell viability was calculated. Cells without sample treatment served as the control group and results were expressed as the percentage of viability of control cells. All experiments were carried out in sextuplicate.

### 2.5. Immunogenicity Assay

In order to investigate the immunogenicity of CHSA/NLS/pDNA, male mice received an injection of CHSA/NLS/pDNA complexes containing plasmid pEGFP-C1 (0.2 mL, *w*/*w* = 15) via the tail vein. The dosage of plasmid was 5 μg per mouse. At 24 h post-injection, the concentrations of interleukin 12 (IL-12) and interferon alpha (IFN-α) in the blood were determined using an ELISA kit according to the manufacturer’s instructions.

### 2.6. Cellular Uptake and Its Mechanisms

To investigate the cellular uptake of CHSA/NLS/pDNA complexes, reporter plasmid pGL3-control was condensed in complexes and CHSA was labeled with FITC. In brief, FITC in anhydrous acetone was added to the CHSA solution (molar ratio of CHSA to FITC was 1:20) and stirred for 24 h in the dark. Excess FITC was removed by dialysis (MWCO 3.5 kDa).

HepG2 and A549 cells were seeded on glass coverslips in 6-well culture plates (1 × 10^5^ cells/well). After growing to 80% confluence, cells were washed twice with PBS and incubated with complexes. After incubating for 4 h, cells were washed with cold PBS, trypsinized, and resuspended in fresh culture medium. Then, cells were filtered using a 35 mm copper wire mesh and measured by flow cytometry (BD Bioscience, Bedford, MA, USA). To investigate the cellular uptake mechanism of CHSA/NLS/pDNA complexes, A549 and HepG2 cells were treated with different membrane entry inhibitors for 1 h at 37 °C. Amiloride (1.5 mM), chlorpromazine (20 μM), methyl-β-cyclodextrin (M-β-CD, 20 μM), sodium azide (NaN_3_, 100 mM), and sucrose (450 μM) were added (1.5 mL per well). In addition, to study the effect of temperature on cellular uptake, cells were incubated with CHSA-3/NLS/pDNA complex at 4 °C for 2 h. Subsequently, cells were washed with precooled PBS three times. Ultimately, cells were collected and the fluorescent intensity of FITC was analyzed using flow cytometry.

### 2.7. Gene Expression In Vitro

#### 2.7.1. pEGFP-C1 Expression Study by LSCM

The transfection efficiency of CHSA/NLS/pDNA complexes was investigated in HepG2 cells. Cells were seeded at a density of 4 × 10^5^ per well in 6-well plates and cultured overnight. The medium was replaced with 2 mL fresh serum-free medium and cells were treated with 100 μL of complex with different formulations at a *w*/*w* ratio of 15: free pDNA, HSA/pDNA, CHSA-2/NLS (scr)/pDNA, CHSA-2/NLS/pDNA, Lipofectamine 2000, and CHSA-3/NLS/pDNA. Each sample contained 500 ng of pEGFP-C1 plasmid. After incubating for 4 h, the serum-free medium was replaced with fresh complete medium and cells were incubated for an additional 24 h at 37 °C. Then cells were washed twice with PBS, treated with Hoechst 33258 (10 μg/mL) at 37 °C for 30 min, and soaked in 4% paraformaldehyde for another 30 min. The transfection and expression of green fluorescence protein were determined using laser scanning confocal microscopy (LSCM; Olympus, Japan).

#### 2.7.2. Luciferase Activity Assay

Cells were seeded in 6-well plates (4 × 10^5^ cells/well) and cultured overnight. The medium was replaced with 2 mL fresh serum-free medium and cells were treated with CHSA-3/NLS/pDNA complexes at different *w*/*w* ratios or different modified complexes at a *w*/*w* ratio of 15. Each sample contained 500 ng of pGL3-control. After 4 h of transfection, the serum-free medium was replaced with fresh complete medium and cells were incubated for an additional 24 h at 37 °C, then washed twice with PBS. The luciferase activity was evaluated using a Luciferase Assay System (E1500, Promega, Madison, WI, USA) and an Infinite 200 Pro luminometer (Tecan, Männedorf, Switzerland).

### 2.8. In Vivo Antitumor Activity

In vivo antitumor activity of CHSA-3/NLS/pDNA containing pcDNA3.0-HA-p53 (CHSA-3/NLS/p53) was investigated in BALB/c nude mice. Tumor-bearing mice were produced by inoculating a suspension of S180 cells (5 × 10^6^ cells in 0.2 mL physiological saline) subcutaneously into the right axillary fossa. The mice were randomly divided into four groups, with six mice in each group, 10 days after inoculation. Mice then received an injection of 0.2 mL of saline, free p53, Lipofectamine, and CHSA-3/NLS/p53 (*w*/*w* = 15%) via the tail vein every day for two weeks. The dosage of pcDNA3.0-HA-p53 plasmid was 0.3 mg/kg per day. Tumor size and body weight of the mice were tested every other day during the treatment period. The mice were sacrificed on the 14th day, and the tumors were removed, measured, and weighed. Tumor volume was calculated with Equation (2):

Tumor volume = (length × width^2^)/2 (2)

The inhibition rate (IR%) was calculated according to Equation (3):

IR (%) = (W_s_ − W_t_)/W_s_ × 100% (3)

where W_s_ and W_t_ represent the tumor weight of the saline group and treatment group, respectively. The p53 protein in tumors was determined by western blotting.

### 2.9. Statistics

All the experiments were repeated at least three times. Results were presented as mean ± standard deviation (SD). Statistical comparisons were performed using a one-way analysis of variance (ANOVA), followed by the Dunnett test. *P*-value < 0.05 was considered statistically significant.

## 3. Results and Discussion

### 3.1. Preparation and Characterization of Cationic Human Serum Albumin

**Figure 2:**
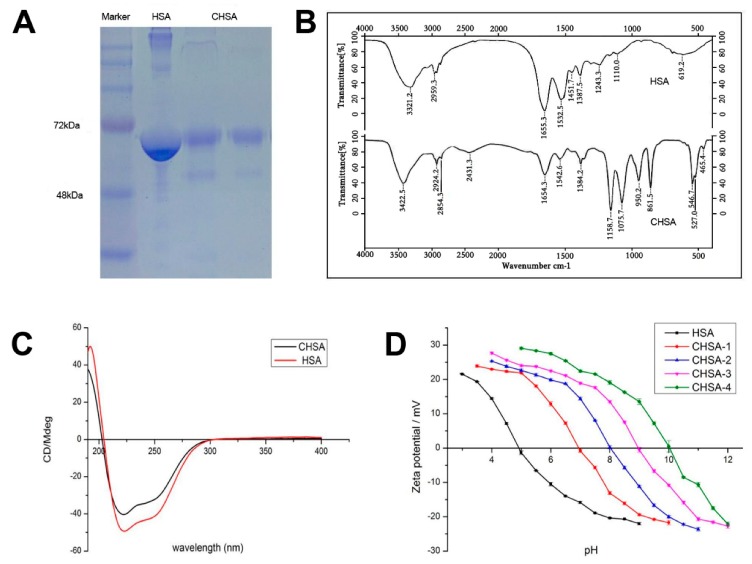
Characterization of cationic human serum albumin (CHSA) by (**A**) SDS-PAGE electrophoresis, (**B**) FTIR spectroscopy, (**C**) circular dichroism, and (**D**) zeta potential and the isoelectric point.

CHSA was obtained through the modification of HSA with ethylenediamine. SDS-PAGE electrophoresis, FTIR spectroscopy, and circular dichroism were used to confirm the characteristics of CHSA. The results are shown in Figure 2. The SDS-PAGE electrophoresis assay showed that the molecular weight of CHSA (about 66 KDa) was slightly higher than that of HSA and there was no distinct dimer in the solution, indicating that the high purity of protein was not influenced by the modification of HSA (Figure 2A). As shown in Figure 2B, the FTIR spectra of CHSA and HSA were not significantly different in the characteristic frequency region (4000–1300 cm^−1^), indicating that there was no obvious distinction in the stretching vibration of the main amide bonds between the two proteins. However, there was a huge difference in the fingerprint area (1300–400 cm^−1^). Strong absorption at 1200–800 cm^−1^ and 500 cm^−1^ was attributed to ν_C–N_ bands in R–CH_2_–NH_2_ and ν_N–H_ bands in –NH_2_. The results revealed that CHSA contained much more –NH_2_ than HSA. The CD spectra of HSA and CHSA are shown in Figure 2C. Both HSA and CHSA exhibited a positive characteristic peak at 192 nm and a negative characteristic at 208 and 222 nm, representing the typical α-helix conformation [22,23]. Results showed that the overall structure of the original protein was maintained during the modification process. Compared with HSA, the positive peak of CHSA at 192 nm and the negative peak at 222 nm decreased slightly, demonstrating that the content of α-helix and β-sheet was reduced in CHSA [24,25]. The result was probably due to the fact that some new amide bonds were synthesized during the reaction, loosening the main peptide chain with a small increase in the β-bend. As a reference carrier, HSA-PEI was synthesized using the primary amines of PEI and HSA-NHS in Supplementary Materials and the product was characterized by FTIR spectrometry (Figure S1).

The zeta potential at different pH values of CHSA with varying degrees of cationization was determined using a dynamic light scattering instrument, and the isoelectric point (pI) of CHSA was obtained according to the pH–zeta potential curve. Figure 2D shows that the zeta potential of HSA and CHSA decreased as pH increased from 1 to 12. The isoelectric point of HSA was approximately 5, while the isoelectric points of CHSA-1, CHSA-2, CHSA-3, and CHSA-4 were 7, 8, 9, and 10, respectively. In addition, the degree of cationization of CHSA was investigated by TNBS, and the results are shown in Table 1.

In this study, nucleic acid molecules were condensed and protected by the positive charges of CHSA, so the alternative CHSA had to show stable positive charges under physiological conditions, as CHSA with an isoelectric point of 10 has so many cations that its half-life in blood circulation is greatly shortened. Meanwhile, CHSA-4 (pI = 4) was more inclined to aggregate with other blood compositions, so it is not suitable as a gene carrier. To investigate the influence of CHSA cationization degree, we selected CHSA-2 (pI = 8) and CHSA-3 (pI = 9) for subsequent experiments. The surface charge of the nanocomplexes prepared by CHSA-2 or CHSA-3 was shifted from negative to slightly positive, which may condense and protect plasmid DNA and thus facilitate high gene-delivery efficiency.

### 3.2. Preparation and Characterization of CHSA/NLS/pDNA Complexes

#### 3.2.1. Preparation of CHSA/NLS/pDNA Complexes

CHSA/NLS/pDNA complexes were prepared with plasmid DNA, nuclear localization signal (NLS) peptide SV40 large T-antigen, and CHSA by electrostatic interaction. NLS can transport macromolecules that cannot pass through the nuclear pore complex, such as plasmid DNA, into the nucleus by active transport. In addition, the positive electrical charge on NLS molecules can interact with the negative charge of pDNA, although the effect is limited. DNA condensation and protection of CHSA/NLS/pDNA complexes were mainly attributed to CHSA.

#### 3.2.2. Hoechst 33258 Intercalation and Gel Retardation Assay

**Figure 3:**
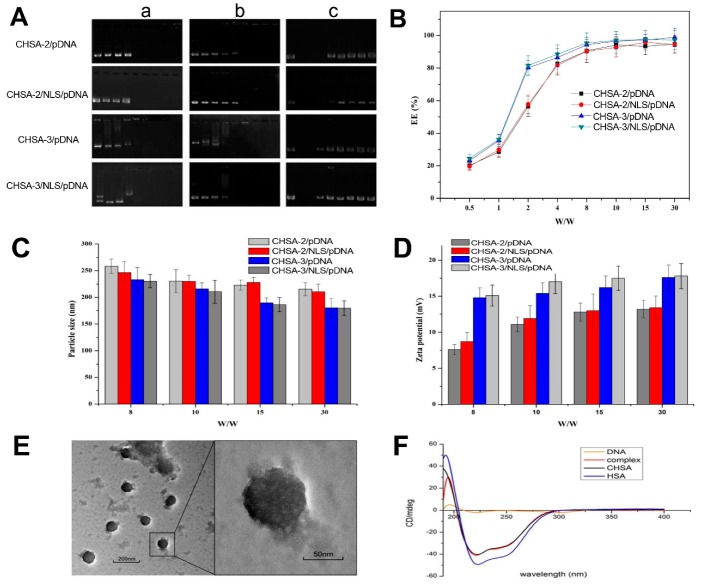
Characterization of CHSA/NLS/pDNA complexes. (**A**) Stability and pDNA protection efficiency of complexes by electrophoresis. Complexes were treated with (a) nothing, (b) heparin, and (c) DNase I. Lanes 1 to 9 correspond to weight ratios of CHSA to pDNA of 0, 0.5, 1, 2, 4, 8, 10, 15, and 30, respectively. (**B**) Encapsulation efficiency, (**C**) particle size, and (**D**) zeta potential of complexes at different *w*/*w* ratios (*n* = 3). (**E**) TEM image and (**F**) CD spectra of CHSA-3/NLS/pDNA complexes at *w*/*w* ratio of 30.

The DNA condensation efficiency of CHSA/NLS/pDNA complexes formed at different weight ratios of CHSA to pDNA were investigated via Hoechst 33258 intercalation and gel retardation assay (Figure 3A,B). As shown in Figure 3B, the encapsulation efficiency of CHSA/NLS/pDNA was *w*/*w* ratio-dependent and exhibited increased encapsulation efficiency as the ratio increased. When the *w*/*w* ratio of CHSA to pDNA was 2, complexes prepared by CHSA-3 exhibited higher encapsulation efficiency (80%) than those prepared by CHSA-2 (60%). This was probably due to the higher isoelectric point of CHSA-3 with more free amino groups, which had a stronger condensation effect on pDNA. However, when the *w*/*w* ratio of CHSA to pDNA was above 8, the encapsulation efficiencies of CHSA-2/pDNA, CHSA-2/NLS/pDNA, CHSA-3/pDNA, and CHSA-3/NLS/pDNA were all above 90% and there was no significant difference between them, revealing that the pDNA in CHSA/pDNA/NLS complexes was fully condensed at a *w*/*w* ratio of 4 (Figure 3B). These results were consistent with those of gel electrophoresis (Figure 3A-a). In addition, HSA-PEI/pDNA complexes were prepared and the DNA condensation efficiency of nanocomplexes formed at different *w*/*w* ratios was analyzed using a Hoechst 33258 intercalation assay. As shown in Figure S2, the encapsulation efficiency of CHSA-pDNA and HSA-PEI/pDNA were *w*/*w* ratio-dependent and exhibited no significant difference.

As shown in Figure 3A, when the *w*/*w* ratio of CHSA-2 to pDNA was above 8, plasmid DNA was not replaced by heparin and complexes were resistant to DNase I digestion, suggesting that CHSA-2 could condense and protect pDNA to some extent. In addition, CHSA-3 was resistant to heparin replacement at a *w*/*w* ratio of 4 and DNase I digestion at *w*/*w* ratio of above 2, which was lower than that of CHSA-2. The results obtained in the Hoechst 33258 intercalation and gel retardation assays showed that complete complexation could be attained when the *w*/*w* ratio was above 4 and CHSA/NLS/pDNA was stable under physiological conditions in the presence of negatively charged protein and nuclease when the N/P ratio was above 8. As is known, one of the challenges in systemic delivery of plasmid DNA is potential degradation of the gene by endonucleases in physiological fluids and the extracellular space. The half-life of plasmid DNA has been estimated to be 10 min following intravenous injection in mice [26]. For this reason, entrapment of plasmid DNA in CHSA/pDNA/NLS complexes was desirable, not only to provide protection from endonuclease degradation but also to improve circulation time.

#### 3.2.3. Particle Size and Zeta Potential Measurement

CHSA/NLS/pDNA complexes prepared at different *w*/*w* ratios were determined by dynamic light scattering. As shown in Figure 3C,D, the particle size and zeta potential of CHSA/NLS/pDNA complexes were *w*/*w* ratio-dependent. As the *w*/*w* ratio increased from 8 to 30, the particle size of CHSA/NLS/pDNA decreased and the zeta potential increased. This suggests that plasmid DNA was condensed by free amino groups on CHSA and the condensation efficiency was dependent on the weight ratio of CHSA to pDNA in the formulation. In addition, complexes prepared by CHSA-3 possessed smaller particle sizes than those prepared by CHSA-2 at the same *w*/*w* ratio, which was probably due to the stronger compression effect of CHSA-3 on pDNA. It is worth mentioning that the particle size of complexes of around 200 nm not only have an enhanced effect on passive targeting of tumor tissues by the EPR effect, but is also beneficial for cellular uptake [27]. Meanwhile, the particle size and zeta potential of CHSA-3/NLS/pDNA and CHSA-3/pDNA were almost the same, suggesting that the addition of NLS peptide had no significant effect on the properties of the complexes. As shown in Figure 3D, the zeta potential of complexes prepared by CHSA-3 was higher than that of complexes prepared by CHSA-2, indicating that CHSA-3/NLS/pDNA may be more stable than CHSA-2/NLS/pDNA under physiological conditions.

#### 3.2.4. Morphology Characterization

The morphology of CHSA-3/NLS/pDNA complexes (*w*/*w* ratio 30) was visually observed via transmission electron microscopy (Figure 3E). The spherical complexes were in well-defined shapes and the complex size was around 50–100 nm, which was relatively smaller than the result obtained by DLS. This is because a large number of hydrophilic groups on the protein surface of CHSA formed a natural hydration film in the aqueous solution, resulting in a larger particle size measured by DLS than by TEM.

#### 3.2.5. Circular Dichroism Analysis

CD spectroscopy can find slight changes in DNA conformation by exploring the differences in absorbance of circularly polarized light by molecules. Circular dichroism results of CHSA-3/NLS/pDNA complexes are shown in Figure 3F. The characteristic peaks of plasmid DNA in complexes disappeared, indicating that the double helix structure of plasmid DNA changed when it was condensed by CHSA. However, the basic secondary structure of CHSA in complexes remained unchanged, showing that CHSA could not only condense and protect pDNA but could also retain the original structure and function to a great extent. These functions are important to achieve optimal biocompatibility as a nonviral gene-delivery system.

### 3.3. In Vitro Cytotoxicity

**Figure 4:**
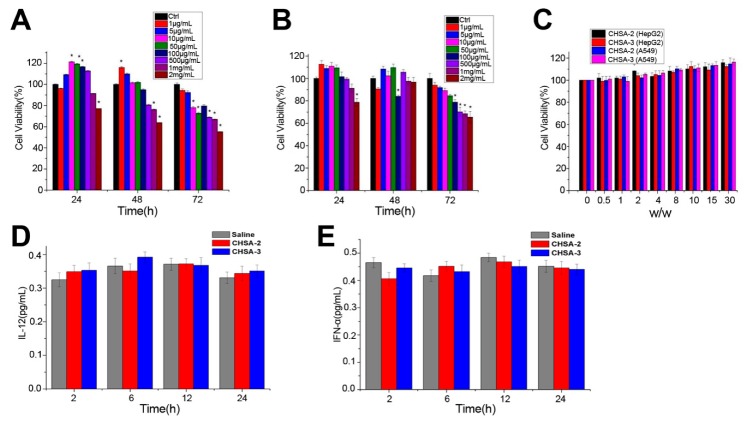
Cytotoxicity in vitro and evaluation of immunogenicity in vivo. In vitro cytotoxicity of CHSA-3/NLS/pDNA complexes with different concentrations of CHSA-3 (1 μg/mL to 2 mg/mL) in (**A**) HepG2 cells and (**B**) A549 cells was determined by MTT assay. (**C**) Cell viability after treating CHSA-3/NLS/pDNA and CHSA-2/NLS/pDNA complexes at various *w*/*w* ratios (0 to 30) for 48 h was also evaluated. Concentration of (**D**) interleukin 12 (IL-12) and (**E**) interferon-alpha (IFN-α) in mice after being injected with CHSA-2/NLS/pDNA and CHSA-3/NLS/pDNA complexes (*w*/*w* 15, CHSA dose 10 mg/mouse). * *p* < 0.05 vs. control group (*n* = 6).

The toxic effects on HepG2 and A549 cells were evaluated by MTT assay after treating CHSA-3/NLS/pDNA complexes with CHSA-3 at different concentrations (1 μg/mL to 2 mg/mL) for 24 h, 48 h and 72 h. As far as we know, HSA is regarded as an endogenous functional protein that plays an important role in the growth and proliferation of cells, such as nutrition, support, and material transport. Previous studies have reported that when CHSA is broken down, the amino acids will provide nutrition to peripheral cells and tissues [7]. As the concentration of CHSA-3 increased, the viability of HepG2 and A549 cells first increased and subsequently decreased (Figure 4A,B), which suggests that CHSA could have a nutritional effect on cells at a low concentration. However, as the concentration reached the milligram level, there was significant cytotoxicity after long-time action. This might be due to the positive charges on the surface of CHSA, which were still cytotoxic to the cells at a large concentration. Nevertheless, the concentration of CHSA was far from cytotoxic under experimental conditions, so CHSA could be considered to be a safe and low-toxicity carrier. In order to explore the influence of the *w*/*w* ratio on cytotoxicity, HepG2 and A549 cells were treated with CHSA-2/NLS/pDNA and CHSA-3/NLS/pDNA complexes at various *w*/*w* ratios for 48 h. As the *w*/*w* ratio increased, the cell viability slightly enhanced (Figure 4C), which was in accordance with the results above, whereas there was no significant difference between the two complexes prepared by CHSA-2 and CHSA-3 and no difference between the two cell types at the same *w*/*w* ratio (*p* > 0.05). In addition, In vitro cytotoxicity of CHSA-3/pDNA and HSA-PEI/pDNA complexes on A549 cells were investigated. As illustrated in Figure S4, HSA-PEI/pDNA showed higher cytotoxicity than CHSA/pDNA, which illustrated that CHSA could be considered as a safe and low-toxicity carrier compared to HSA-PEI.

### 3.4. Immunogenicity Study

For practical application, the safety of the delivery system is the most important issue and must be strictly evaluated, as it will inevitably contact various biomacromolecules, organelles, cells, and tissues when used in living systems [28]. As previously reported, IL-12, a 70 kDa heterodimeric cytokine, was demonstrated to activate inflammatory and immune responses. IFN-α, a glycoprotein secreted by leukocytes and fibroblasts, is considered to exert immunomodulatory effects [29]. Therefore, IL-12 and IFN-α can be considered as indicators to detect immunogenicity. After intravenous injection, the concentrations of IL-12 and IFN-α in blood were detected. As shown in Figure 4D,E, compared to the saline group, no significant difference was observed in complexes prepared with CHSA-2 and CHSA-3. As far as we know, the IL-12 family of cytokines acts as an immunological leader, shaping immune responses by directly inducing the development of T cells and altering the function of many cell populations that bring disease results [30]. Our results also showed that CHSA-based complexes had a significantly lower immunogenicity and lower hemolytic toxicity than complexes prepared with cationic polymer polyetherimide conjugated human serum albumin PEI-HSA (Figures S3 and S6). The results indicated that CHSA had very low if any immunogenicity as a gene carrier. From in vitro cytotoxicity and in vivo immunogenicity studies, it is believed that CHSA-based complexes are likely to be well tolerated in vivo without any deleterious side effects.

### 3.5. Cellular Uptake and Its Mechanisms

**Figure 5:**
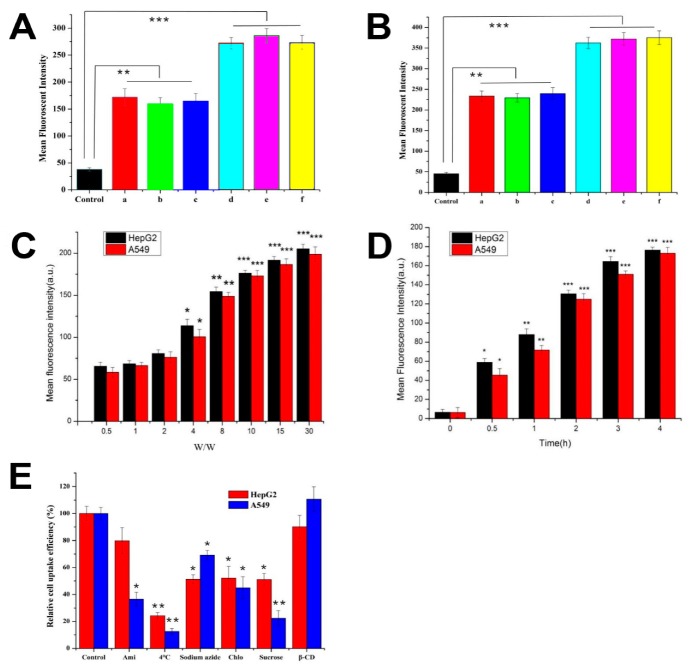
Cellular uptake and its mechanism. Cell uptake of different complexes (a: CHSA-2/pDNA; b: CHSA-2/NLS(scr)/pDNA; c: CHSA-2/NLS/pDNA; d: CHSA-3/pDNA; e: CHSA-3/NLS(scr)/pDNA; f: CHSA-3/NLS/pDNA) was investigated in (**A**) HepG2 cells and (**B**) A549 cells by determining mean fluorescent intensity by flow cytometry (*w*/*w* 15). Influence of (**C**) *w*/*w* ratio and (**D**) time intervals on transfection efficiency of CHSA-3/NLS/pDNA complexes (*w*/*w* 15) was evaluated. (**E**) Effect of inhibitors on internalization of CHSA-3/NLS/pDNA complexes (*w*/*w* 15) was investigated in HepG2 and A549 cells. Results are expressed as mean ± SD (*n* = 6). * *p* < 0.05, ** *p* < 0.01, *** *p* < 0.001 vs. control group (*n* = 6).

To study the cellular uptake of CHSA/NLS/pDNA complexes, HepG2 and A549 cells were incubated with FITC-labeled complexes. The mean fluorescence intensity of complexes were evaluated by flow cytometry. As shown in Figure 5A,B, CHSA-3/NLS/pDNA showed stronger cellular uptake than CHSA-2/NLS/pDNA in both cells at a *w*/*w* ratio of 15 (*p* < 0.05). The results suggest that CHSA-3, which has more positive surface charges, could play a greater role in improving cellular uptake. It is also noteworthy that there was no significant difference between CHSA-2/pDNA and CHSA-2/NLS/pDNA (*p* > 0.05), so we were able to conclude that the introduction of NLS peptide had no significant effect on cellular uptake. In addition, the mean fluorescence intensity of CHSA-3/NLS/pDNA complexes was enhanced in both cells as the *w*/*w* ratio was gradually increased (Figure 5C). With a *w*/*w* ratio less than 2, no significance was observed between CHSA-3/NLS/pDNA complexes and the control group, but when the *w*/*w* ratio was up to 4, cellular uptake increased significantly. Most probably, CHSA was internalized into the cells through the positively charged amino groups combined with the negatively charged proteins on the cell surface and the increment slowed down while the combination tended to be saturated. Moreover, as shown in Figure 5D, the mean fluorescence intensity was gradually enhanced over time.

Understanding the uptake mechanism of nonviral gene delivery systems is important in the development of more efficient carriers. An endocytosis inhibition assay was carried out in order to explore the endocytosis pathway for CHSA/NLS/pDNA complexes. As shown in Figure 5E, the cellular uptake efficiency of complexes was significantly decreased after treatment with low temperature (down to 18% in A549 cells, *p* < 0.01) and NaN_3_ (down to 54% in HepG2 cells, *p* < 0.05). It is widely known that low temperature can inhibit cell metabolism, thereby reducing the energy produced in cells. Meanwhile, NaN_3_ combined with 2-deoxyglucose can interfere with the glycolysis and oxidative metabolism pathway, thereby blocking the production of adenosine triphosphate (ATP) in cells [31]. The results indicate that the internalization of complexes was clearly energy-dependent. As is known, high concentrations of sucrose can inhibit cell endocytosis in a hypertonic environment. After incubation with sucrose, the cellular uptake rate in both cells decreased significantly (down to 34.6% in HepG2 cells, *p* < 0.05, and 20.3% in A549 cells, *p* < 0.01), indicating that the complexes entered these two cells through the endocytosis pathway. In addition, M-β-CD can extract cholesterol from the cell membrane and inhibit caveolin-mediated endocytosis [12]. Compared to the untreated group, no significant change was found after treatment with M-β-CD, suggesting that the caveolin-mediated endocytosis pathway was not the main route into cells. At the same time, the cellular uptake rate was significantly reduced after treatment with chlorpromazine (down to 45% on average, *p* < 0.05), showing clathrin-dependent endocytosis. Moreover, after amiloride treatment, no obvious difference (*p* > 0.05) was found in HepG2 cells, while there was a distinct decrease in A549 cells (down to 34.6%, *p* < 0.05). The results indicated that amiloride exerted an effect on the uptake of complexes in A549 cells. The Na^+^/H^+^ antiporter on the surface of the cell membrane is an indispensable part of the macropinocytosis pathway. Amiloride can block the Na^+^/H^+^ antiporter and eventually block the macropinocytosis pathway [32]. Therefore, the macropinocytosis pathway is one way that complexes can enter A549 cells. This was probably because there were many positive amino groups on the surface of CHSA that could interact with negatively charged proteins on the cell surface and stimulate the cell to upregulate micropinocytosis. Cellular uptake is a complicated process, as it involves many molecules, and sometimes the same kind of complex can be internalized through different uptake pathways in different cell types, or sometimes the same type of cell can take up different kinds of complexes through different mechanisms [31]. Some proteins, such as small GTPase and Ras-related to the brain (Rab), are essential for the macropinocytosis process [33]. The results here may be due to the different levels of proteins in the macropinocytosis process between A549 and HepG2 cells. In general, the aforementioned results have illustrated that the internalization of CHSA/NLS/pDNA complexes in HepG2 cells is mainly by the clathrin-dependent endocytosis pathway. By contrast, complexes entered A549 cells by the macropinocytosis- and clathrin-dependent endocytosis pathways.

### 3.6. Gene Expression In Vitro

**Figure 6:**
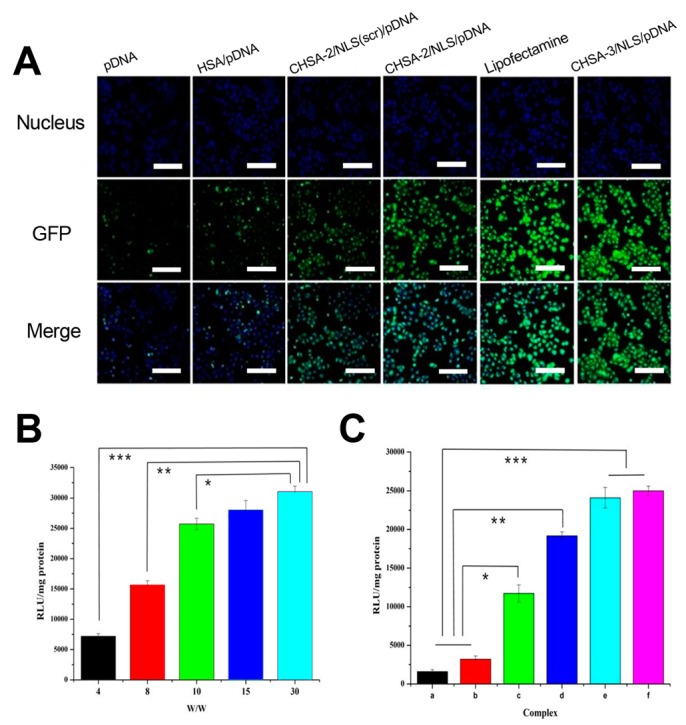
In vitro gene expression in HepG2 cells analyzed by laser scanning confocal microscopy (LSCM) and chemiluminescence. (**A**) Green fluorescent protein (GFP) expression after 24 h of transfection with pDNA alone, HSA/pDNA, CHSA-2/NLS (scr)/pDNA, CHSA-2/NLS/pDNA, Lipofectamine 2000, and CHSA-3/NLS/pDNA (*w*/*w* 15, scale bar in all pictures indicates 200 μm). Luciferase activity of cells transfected with (**B**) CHSA-3/NLS/pDNA at different *w*/*w* ratios and (**C**) modified complexes at a *w*/*w* ratio of 15 for 24 h was examined (a: pDNA alone; b: HSA/pDNA; c: CHSA-2/NLS(scr)/pDNA; d: CHSA-2/NLS/pDNA; e: Lipofectamine 2000; f: CHSA-3/NLS/pDNA). Results are expressed as mean ± SD (*n* = 6). * *p* < 0.05, ** *p* < 0.01, *** *p* < 0.001.

In vitro gene expression of green fluorescent protein (GFP) was qualitatively analyzed by LSCM. Gene expression of different delivery carriers such as free pDNA, HSA/pDNA complexes, Lipofectamine, and CHSA/NLS/pDNA complexes was evaluated. In addition, a scrambled sequence NLS(scr) was introduced to analyze the nuclear locating function of NLS. As shown in Figure 6A, GFP expression in cells treated with free pDNA and HSA/pDNA was negligible, while the fluorescence in cells treated with CHSA/NLS/pDNA complexes was enhanced. It was obvious that the fluorescence intensity of CHSA-2/NLS/pDNA showed higher gene expression efficiency than CHSA-2/NLS(scr)/pDNA, showing that NLS peptide played an important role in gene expression. This result was probably due to NLS interacting with the nucleus transport system and consequently promoting the nuclear import of pDNA. Results showed that CHSA-3/NLS/pDNA could induce higher gene expression efficiency than CHSA-2/NLS/pDNA and was similar to that of Lipofectamine 2000 and PEI-HAS (Figure S5).

To further confirm the gene-delivery ability, cells were transfected with CHSA/NLS/pDNA complexes containing pGL3-control plasmid, and luciferase activity was measured by chemiluminescence. As shown in Figure 6B,C luciferase activity increased significantly as the *w*/*w* ratio of CHSA-2/NLS/pDNA complexes increased from 4 to 30. In accordance with the transfection results above, the fluorescence intensity of CHSA-2/NLS/pDNA was higher than that of CHSA-2/NLS(scr)/pDNA and lower than that of CHSA-3/NLS/pDNA (*p* < 0.05). Moreover, the fluorescence intensity of CHSA-3/NLS/pDNA was slightly stronger than that of Lipofectamine 2000, although the two groups showed no statistically significant difference (*p* > 0.05). These results suggest that CHSA/NLS/pDNA could significantly enhance gene expression efficiency, thereby demonstrating CHSA/NLS/pDNA as an effective gene-delivery system for plasmid DNA.

### 3.7. In Vivo Antitumor Activity

**Figure 7:**
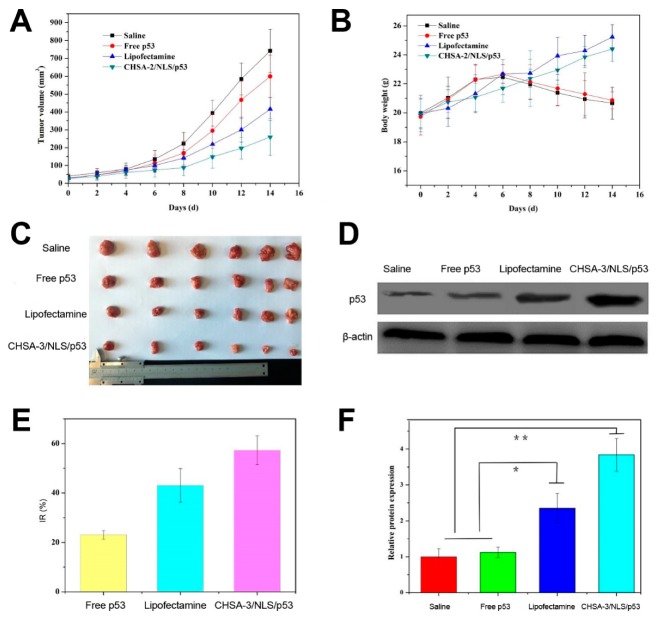
In vivo antitumor activity. (**A**) Tumor volume and (**B**) body weight of S180 xenografted BALB/c nude mice after intravenous administration of saline, free p53 plasmid, Lipofectamine containing p53 plasmid, and CHSA-3/NLS/p53 complexes. (**C**) Tumor graph and (**D**) western blotting image. (**E**) Tumor inhibition rate (IR%) and (**F**) relative protein expression were calculated and analyzed. * *p* < 0.05, ** *p* < 0.01 (*n* = 6).

To determine whether in vitro gene expression data are translatable to in vivo settings, we examined the in vivo antitumor activity of CHSA-3/NLS/pDNA complexes. To survey the tumor-suppressing ability of the complexes, we utilized plasmid pcDNA3.0-HA-p53 as a therapeutic gene. CHSA-3/NLS/pDNA complexes containing pcDNA3.0-HA-p53 (CHSA-3/NLS/p53) were investigated on S180 xenografted BALB/c nude mice. As shown in Figure 7A,C the tumor volume of the CHSA-3/NLS/p53 group was significantly smaller than that of saline, free plasmid, and even commercial reagent Lipofectamine groups. At the same time, the body weight of CHSA-3/NLS/p53 and Lipofectamine-treated mice increased, while mice treated with saline and free plasmid lost weight after the eighth day of administration (Figure 7B). As far as we know, changes in body weight are regarded as an indication of safety, so the results more likely indicated the outstanding compatibility of CHSA-3/NLS/p53. Similar results were observed in the inhibition rate (IR%), as shown in Figure 7E. Compared with saline, free plasmid displayed relatively low antitumor activity (IR% = 23.1%), while CHSA-3/NLS/p53 had the highest inhibition rate (57.3%). Most probably, naked pDNA would be readily degraded in the blood circulation and consequently have an indistinct effect on tumors. This contrast also shows that CHSA-3/NLS/DNA complexes could escape capture by the RES system during blood circulation to some extent and arrive at the tumor site to suppress tumor growth.

Tumor suppressor protein p53 in tumor tissue of S180 xenografted mice was determined by western blotting, and the results are shown in Figure 7D,F. There was significant upregulation of the p53 protein after treatment with CHSA-3/NLS/p53 complexes, implying that the antitumor activity was more likely due to the high expression level of the p53 protein.

## 4. Conclusions

In this study, a simple and effective CHSA-based gene delivery system was successfully constructed and evaluated. CHSA/NLS/pDNA complexes showed low immunogenicity and high pDNA encapsulation efficiency, while they were able to condense and protect pDNA against degradation by nuclease in blood circulation and clearance by the RES system in vivo. In addition, complexes displayed a nutritional effect on cells at low concentration and no significant cytotoxicity. Furthermore, CHSA/NLS/pDNA complexes exhibited admirable cellular uptake and gene expression efficiency, with the help of a nuclear localization signal. More importantly, CHSA/NLS/p53 displayed desired in vivo antitumor ability. All these results have enhanced our confidence that CHSA/NLS/DNA could be a new strategy for gene therapy.

## Figures and Tables

**Table 1:** Numbers of free amino groups (FAGs) for CHSA with different isoelectric points (pI) (*n* = 3).

	Material	HSA	CHSA-1	CHSA-2	CHSA-3	CHSA-4
Character						
pI		5	7	8	9	10
FAG		–	22.09 ± 3.52	32.71 ± 1.37	42.51 ± 3.11	57.10 ± 2.49

## Supplementary Materials

The following are available online at https://www.mdpi.com/1999-4923/11/11/608/s1, Figure S1. Characterization of HSA-PEI by FTIR spectrometry, Figure S2. Encapsulation efficiency of CHSA/pDNA and HSA-PEI/pDNA complexes with a series of *w*/*w* ratios by Hoechst 33258 intercalation assay, Figure S3. The hemolysis of CHSA/pDNA and HSA-PEI/pDNA complexes (*w*/*w* ratio = 15) at different concentrations of plasmid after incubation for 4 h, Figure S4. In vitro cytotoxicity study of CHSA/pDNA and HSA-PEI/pDNA complexes (*w*/*w* 15) at different time point against A549 cells. * *p* < 0.05, Figure S5. Luciferase activity of A549 cells transfected with CHSA/pDNA and HSA-PEI/pDNA complexes (*w*/*w* 15) for 24 h. n.s. indicates no significance, Figure S6. Concentration of (A) interferon-alpha (IFN-α) and (B) interleukin 12 (IL-12) in the blood of mice after being injected with CHSA/pDNA and HSA-PEI/pDNA complexes. * *p* < 0.05, ** *p* < 0.01.
