# Supplementary material for: An Effective Cationic Human Serum Albumin-Based Gene-Delivery Carrier Containing the Nuclear Localization Signal
Source: Pharmaceutics. 2019 Nov 13;11(11):608. doi: 10.3390/pharmaceutics11110608 (PMC6920835; doi:10.3390/pharmaceutics11110608)
Supplement: Supplementary file 1 [file pharmaceutics-11-00608-s001.pdf]

pharmaceutics

Supplementary Materials: An Effective Cationic Human Serum Albumin–Based Gene-Delivery Carrier Containing the Nuclear Localization Signal

Guannan Guan, Baohui Song, Jie Zhang, Kang Chen, Haiyang Hu, Mingyue Wang and Dawei Chen

1. Methods

1.1. Preparation of HSA-PEI and HSA-PEI/pDNA Complexes

EDC (1-ethyl-(3-3-dimethylaminopropyl) carbodiimide hydrochloride) and NHS (*N*-hydroxy succinimide) were used to connect HSA and PEI (obtained from Sigma-Aldrich, branched, average Mw ~25,000 by LS, average Mn ~10,000 by GPC) as described previously. Briefly, 10 mg of HSA was dissolved in a pH 4 2-morpholinoethanesulfonic acid (MES)/NaCl buffer which consisted of 0.05 M MES and 0.5 M NaCl. 300 mg of EDC and 0.06 M NHS in MES/NaCl buffer were added to form ester of NHS–HSA. 10 mg of PEI reacted with the solution of NHS–HSA under the gentle stir by magnetic stirrer for 24 h in room temperature. The final product, HSA-PEI, was purified by dialysis (MWCO 20 kDa) against deionized water for 48 h and lyophilization. Then HSA-PEI were characterized by FTIR.

HSA-PEI/pDNA complexes were prepared with plasmid DNA and HSA-PEI by electrostatic interaction. HSA-PEI was mixed with pDNA at a series of *w*/*w* ratios (weight ratio of HSA-PEI to pDNA) in phosphate buffer solution (PBS, pH 7.4) for 30 min at room temperature. The DNA condensation efficiency of nanocomplexes formed at different *w*/*w* ratios was analyzed using a Hoechst 33258 intercalation assay. Plasmid pGL3-control and CHSA(pI = 8) was used in the following experiments.

1.2. Hemolytic Test

The hemolytic effect of HSA-PEI was evaluated using New Zealand rabbit erythrocytes. In brief, the plasma samples were centrifuged and diluted with normal saline to separate the red blood cells (RBCs 2%). Afterwards, 2 mL of red blood cells was added to each sample. As a comparison, 2 mL of normal saline (negative control) and 2 mL of distilled water (positive control) were added. HSA-PEI and CHSA/pDNA (0.5 mL respectively, *w*/*w* ratio = 15) at various concentration of plasmid (10, 50, 100, 200, 400 and 800 μg/mL) were incubated with a 2 mL RBC suspension, and proper normal saline was added to maintain the same volume. All samples were kept at 37 °C for 4 h and then centrifuged at 5000 rpm for 10 min. The absorbance of the supernatant was measured using a UV spectrophotometer at 570 nm. The hemolysis percentage of RBCs was determined using the following equation:

$$\text{Hemolysis ratio (\%)} = (A_1 - A_0) / (A_2 - A_0) \times 100\%$$

where A_0, A_1 and A_2 represent the absorbance of negative control, samples and positive control, respectively.

1.3. MTT Assay

A549 cells were cultured in DMEM supplemented with 10% FBS and 1% antibiotics (penicillin/streptomycin) at 37 °C in a humidified atmosphere containing 5% CO_2. Cells were seeded in 96-well plates (1×10^4 cells/well) and cultured overnight. Cells were treated with CHSA/pDNA and HSA-PEI/pDNA complexes at a *w*/*w* ratio of 15 for 24 h, 48 h and 72 h. After cells were incubated, 10 μL MTT (5 mg/mL) was added to each well. The cells were further incubated for 4 h, then 150 μL

DMSO was added to dissolve the formazan crystals that formed in the live cells. Absorbance of the samples was measured at 490 nm and cell viability was calculated. Cells without sample treatment served as the control group and results were expressed as percentage of viability of control cells.

1.4. Luciferase Gene Expression In Vitro

A549 cells were seeded in 6-well plates (4 × 10^5 cells/well) and cultured overnight. The medium was replaced with 2 mL fresh serum-free medium and cells were treated with CHSA/pDNA and HSA-PEI/pDNA at a *w/w* ratio of 15, respectively. Each sample contained 500 ng of pGL3-control. After 4 h of transfection, the serum-free medium was replaced with fresh complete medium and cells were incubated for an additional 24 h at 37 °C, then washed twice with PBS. The luciferase activity was evaluated using a Luciferase Assay System (E1500, Promega, USA) and an Infinite 200 Pro luminometer (Tecan, Switzerland).

1.5. Immunogenicity Assay

In order to investigate the immunogenicity of CHSA/pDNA and HSA-PEI/pDNA, mice received an injection of complexes containing plasmid pGL3-control (0.2 mL, *w/w* 15) via the tail vein. The dosage of plasmid was 5 μg per mouse. At 24 h post-injection, the concentrations of interleukin 12 (IL-12) and interferon alpha (IFN-α) in the blood were determined using an ELISA kit according to the manufacturer's instructions.

2. Results and Discussion

2.1. Preparation of HSA-PEI and HSA-PEI/pDNA Complexes

HSA-PEI was synthesized using the primary amines of PEI and HSA-NHS, obtained by activating the carboxyl groups of HSA with NHS. The product was characterized by FTIR spectrometry. The absorbance peak of the amino group was presented in 3460.3 cm^{-1} by PEI; that of amide bond was presented in 1571.7 cm^{-1} and 1,649.0 cm^{-1}. The three absorbance peaks appeared in the FTIR spectrometry of HSA-PEI, indicating acylation of the amino group of PEI with HSA.

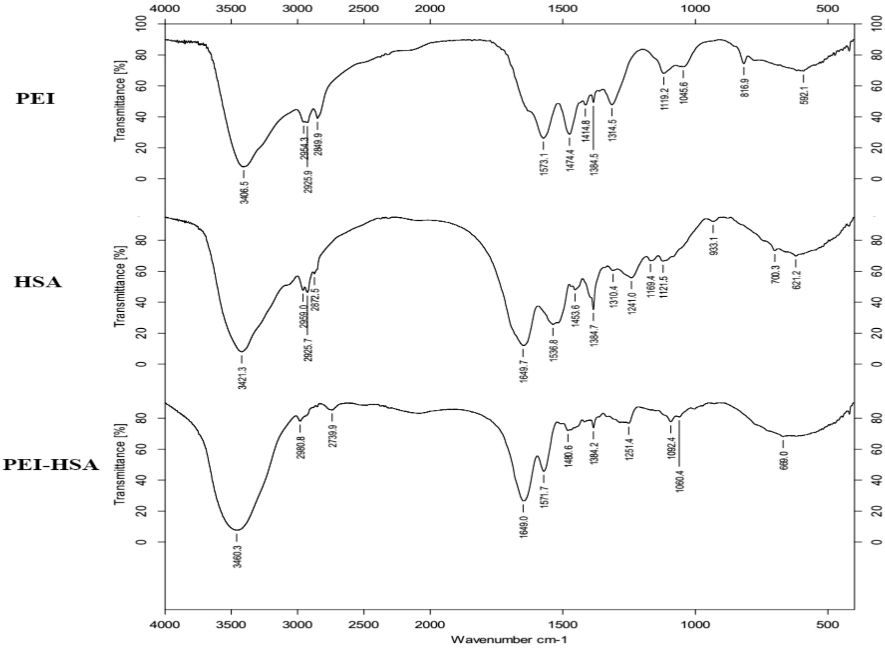

Figure S1. Characterization of HSA-PEI by FTIR spectrometry.

HSA-PEI/pDNA complexes were prepared and the DNA condensation efficiency of nanocomplexes formed at different *w/w* ratios was analyzed using a Hoechst 33258 intercalation assay. As shown in Figure S2, the encapsulation efficiency of CHSA-pDNA and HSA-PEI/pDNA were *w/w* ratio-dependent and exhibited no significant difference.

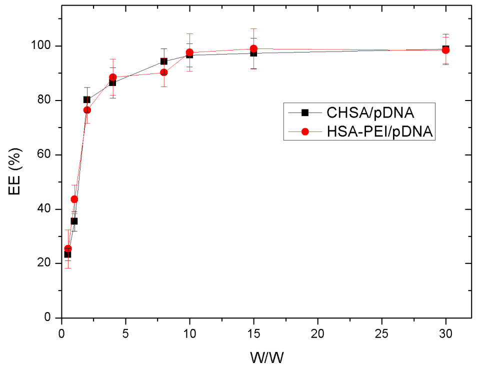

Figure S2. Encapsulation efficiency of CHSA/pDNA and HSA-PEI/pDNA complexes with a series of *w*/*w* ratios by Hoechst 33258 intercalation assay.

2.2. Hemolytic Test

One of the obstacles to in vivo application of cationic polymers is the nonspecific interactions with blood components. The compatibility of cationic vectors with blood is very important for its introduction into the systemic circulation. Hence, the hemolytic effect of CHSA/pDNA and HSA-PEI/pDNA complexes was evaluated through an in vitro hemolytic assay. As shown in Figure S3, the hemolysis rate of HSA-PEI/pDNA gradually enhanced when the concentration increased from 10 to 800 μg/mL, suggesting they had strong hemolytic toxicity. Nevertheless, the hemolytic toxicity of CHSA/pDNA was relatively low.

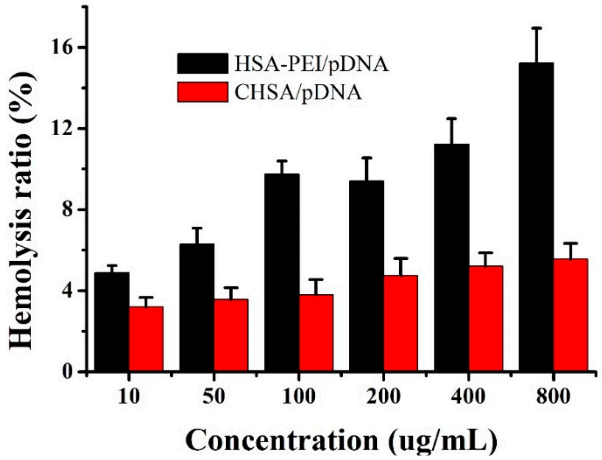

Figure S3. The hemolysis of CHSA/pDNA and HSA-PEI/pDNA complexes (*w*/*w* ratio = 15) at different concentrations of plasmid after incubation for 4 h.

2.3. MTT Assay

In vitro cytotoxicity of CHSA/pDNA and HSA-PEI/pDNA complexes on A549 cells were investigated. As illustrated in Figure S4, HSA-PEI/pDNA showed higher cytotoxicity than CHSA/pDNA. Cell viability of CHSA/pDNA group was still over 80% at 72 h while cells treated with HSA-PEI/pDNA complexes showed less than 70% viability. CHSA could be considered as a safe and low-toxicity carrier compared to HSA-PEI.

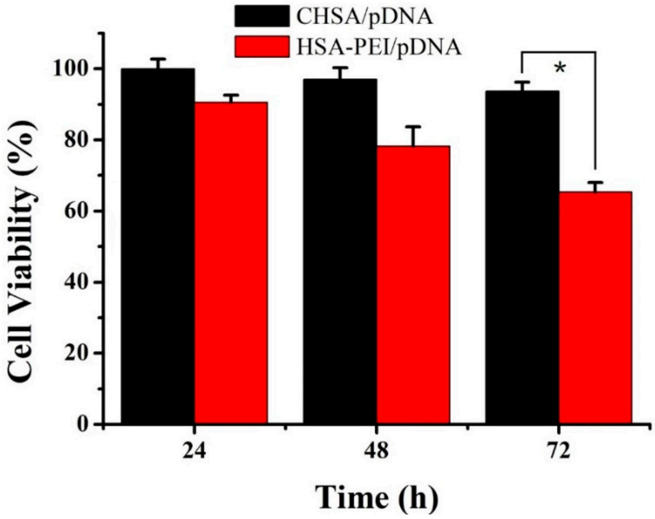

Figure S4. In vitro cytotoxicity study of CHSA/pDNA and HSA-PEI/pDNA complexes (*w*/*w* 15) at different time point against A549 cells. * $p < 0.05$.

2.4. Luciferase Gene Expression In Vitro

Luciferase gene expression in vitro in A549 cells was evaluated to compare CHSA/pDNA and HSA-PEI/pDNA complexes. Results in Figure. S5 showed there was no significant difference between CHSA/pDNA and HSA-PEI/pDNA in Luciferase gene expression in vitro at 24 h post transfection.

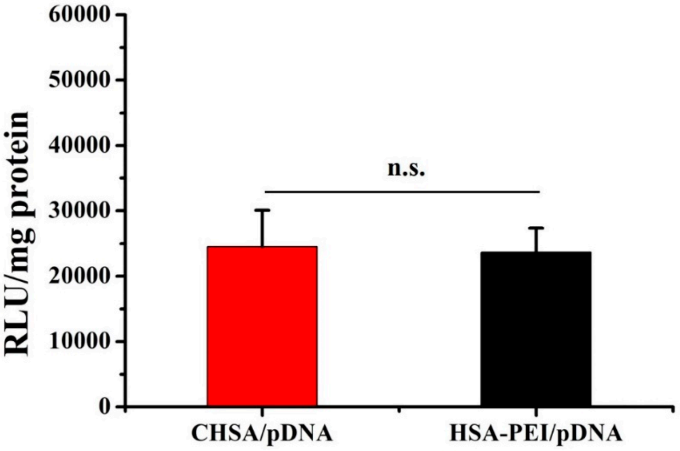

Figure S5. Luciferase activity of A549 cells transfected with CHSA/pDNA and HSA-PEI/pDNA complexes (*w*/*w* 15) for 24 h. n.s. indicates no significance.

2.5. Immunogenicity Assay

IL-12 and IFN-α were determined as indicators to detect immunogenicity. After intravenous injection, the concentrations of IL-12 and IFN-α in blood were detected. As shown in Figure S6, compared to the saline group, no significant difference ($p > 0.05$) was observed in complexes prepared with CHSA, while HSA-PEI/pDNA exhibited high secretion level of IL-12 and IFN-α. The results indicated that CHSA had lower immunogenicity than HSA-PEI.

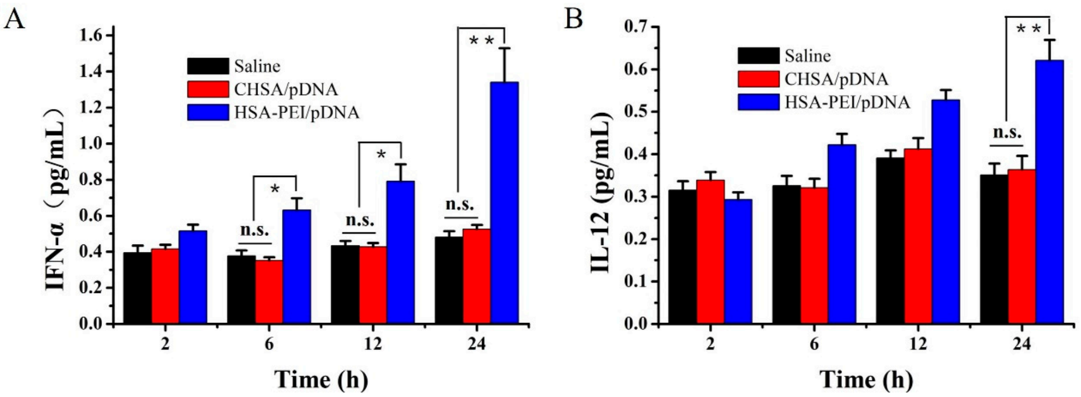

Figure S6. Concentration of (**A**) interferon-alpha (IFN-α) and (**B**) interleukin 12 (IL-12) in the blood of mice after being injected with CHSA/pDNA and HSA-PEI/pDNA complexes. * $p < 0.05$, ** $p < 0.01$.

3. Conclusions

In this supplementary file, the hemolytic effect and cytotoxicity in vitro as well as immunogenicity of CHSA/pDNA and HSA-PEI/pDNA complexes were investigated to illustrate the difference between cationic human serum albumin and previously reported PEI-HSA. Results shows that CHSA/pDNA had lower hemolysis ratio and cytotoxicity as well as lower immunogenicity than HSA-PEI/pDNA complexes at the same *w*/*w* ratio. In addition, there was no significant difference between HSA-PEI/pDNA and CHSA/pDNA in the luciferase gene expression in A549 cells in vitro. From the results above, we can draw the conclusion that CHSA/pDNA complexes are better than HSA-PEI/pDNA complexes as safe and effective gene carrier.
